# Left atrial inflow propagation velocity derived by color M-mode Doppler in acute heart failure

**DOI:** 10.1007/s10554-022-02614-y

**Published:** 2022-04-23

**Authors:** Øyvind Johannessen, Peder L. Myhre, Brian Claggett, Moritz Lindner, Eldrin F. Lewis, Jose Rivero, Susan Cheng, Elke Platz

**Affiliations:** 1 Department of Cardiology, Division of Medicine, Akershus University Hospital, Lørenskog, Norway; 2 Faculty of Medicine, Institute of Clinical Medicine, University of Oslo, Oslo, Norway; 3 Cardiovascular Division, Brigham and Women’s Hospital, Boston, USA; 4 Harvard Medical School, Boston, USA; 5 Zentralklinik Bad Berka, Bad Berka, Germany; 6 Cardiovascular Division, Stanford University, San Francisco, USA; 7 Department of Cardiology, Cedars-Sinai Medical Center, Los Angeles, USA

**Keywords:** Acute heart failure, Color M-mode Doppler, Echocardiography, Left atrial function, Pulmonary veins

## Abstract

Left atrial (LA) inflow propagation velocity from the pulmonary vein (LAIF-PV) has been proposed as a novel measure of LA reservoir function and is associated with pulmonary capillary wedge pressure in critically ill patients. However, data on LAIF-PV in acute heart failure (AHF) are lacking. We sought to examine the feasibility of measuring LAIF-PV and evaluate clinical and echocardiographic correlates of LAIF-PV in AHF. In a prospective cohort study of adults hospitalized for AHF, we used color M-mode Doppler of the pulmonary veins to obtain LAIF-PV in systole. Among 142 patients with appropriate images and no more than moderate mitral regurgitation, LAIF-PV measures were feasible in 76 patients (54%) aged 71 ± 14 years, including 68% men with left ventricular ejection fraction (LVEF) 38% ± 13. Mean LAIF-PV was 24.2 ± 5.9 cm/s. In multivariable regression analysis adjusted for age, sex, systolic blood pressure, heart rate, body mass index, New York Heart Association class, LA volume and LVEF, the only independent echocardiographic predictors of LAIF-PV were right ventricular (RV) S’ [ß 0.46 cm/s per cm/s (95% CI 0.01–0.91), p = 0.045] and tricuspid annular plane systolic excursion (TAPSE) [ß 0.28 cm/s per mm (95% CI 0.02–0.54), p = 0.039]. Notably, LAIF-PV was not significantly correlated with measures of LV function, LA function or E/e’. In conclusion, LAIF-PV was measurable in 54% of patients with AHF, and lower values were associated with measures of impaired RV systolic function but not LV or LA function.

**Supplementary information:**

The online version contains supplementary material available at 10.1007/s10554-022-02614-y.

## Introduction

Left atrial (LA) size and function have gained interest as diagnostic and prognostic imaging markers in patients with atrial fibrillation, mitral valve disease, hypertension, ischemic heart disease, and heart failure (HF) [1–4]. Estimating intracardiac pressures, particularly LA pressure, is valuable for diagnosis, monitoring disease progression, and evaluating treatment effect in chronic and acute HF (AHF) [5]. Although invasive measures of capillary wedged pressure (PCWP) by right heart catheterization is the gold standard for assessing LA pressures and function, this procedure is not routinely performed in AHF given trials failing to show improved outcomes with invasive measurements of intracardiac pressures compared to non-invasive measurements [6, 7]. Thus, invasive intracardiac pressure measurements are reserved for select subgroups of AHF such as non-responders to medical therapy or cardiogenic shock [8].

In routine clinical practice, cardiologists are required to rely on non-invasive surrogates to estimate left ventricular (LV) filling pressures. Such surrogates include echocardiographic (TTE) measures of early diastolic mitral flow divided by mitral annulus tissue movement (E/e’) and LA size and function [9]. In addition, lung ultrasound (LUS) assessments of B-line artifacts have been identified as a sensitive approach for quantifying pulmonary congestion and are included in the diagnostic algorithm for AHF [10].

The combination of Color flow and M-mode (color M-mode Doppler) allows for real-time evaluation of the blood flow propagation across an orifice or a valve. This mode is typically used over the mitral valve and provides information about LV diastolic function [11]. Recently, LA inflow propagation velocity (LAIF-PV) measured by Color M–mode Doppler of the pulmonary veins was proposed as a novel, non-invasive measure of LA reservoir function [13], and an independent estimate of PCWP [12, 13].

Whether LAIF-PV is a clinically useful measurement in AHF remains unknown. We sought to assess the feasibility of LAIF-PV in patients with AHF, and to determine clinical, hemodynamic, and echocardiographic correlates of LAIF-PV. In exploratory analyses, we also aimed to assess LAIF-PV in association with long-term clinical outcomes. We hypothesized that increased LAIF-PV was associated with clinical and echocardiographic measures related to congestion.

## Methods

### Patient population

We analyzed data from a prospective, observational study that enrolled 196 adult patients admitted to an academic hospital with AHF, between April 2015 and August 2017. Details regarding the design of this study have been published previously [14]. For the purpose of this analysis we excluded patients with aortic and/or mitral valve repair or prosthesis, moderate to severe mitral regurgitation, significant pulmonary disease (e.g. cancer, fibrosis, and pneumonia) and large pericardial effusion. Clinical, demographic and laboratory data were extracted from the patients’ hospital records by trained investigators. We included patients with AF during echocardiography as the main objective was to assess the feasibility of this technique. The study complied with the Declaration of Helsinki and was approved by the local institutional review board. Written informed consent was obtained from all study participants.

### Measurements by echocardiography and lung ultrasound

All patients underwent comprehensive TTE and LUS exams early during admission. TTEs were performed with 2–5 MHz phased array transducers and standard ultrasound equipment from three vendors (General Electric, Milwaukee, Wisconsin; Phillips, Bothell, Washington; and Siemens, Erlangen, Germany). TTE cine loops were analyzed offline with echocardiographic software (Syngo Dynamics; Siemens, Malvern, Pennsylvania). Image acquisition and analysis of cardiac structure and function was performed by one of two investigators, who were blinded to other patient data, including LUS results. Echocardiographic measurements were performed according to current American Society of Echocardiography guidelines [15, 16].

LA minimum volume (LAV_min_) and LA maximum volume (LAV_max_) were measured by the biplane Simpson method of disks at the end of LV diastole and end of LV systole, respectively [15]. LA volume was indexed to body surface area to estimate LA volume index (LAVi). LA emptying fraction (LAEF) and LA expansion index (LAEI) are relevant indices of LA reservoir function [1, 17]. LAEF was calculated as the ratio of LA blood volume ejected from LA during LV diastole (LAV_max_ – LAV_min_) relative to volume in the LA at the end of systole (LAV_max_)) using the formula:

$${\text{LAEF = [(LAV}}_{{\max }} - {\text{LAV}}_{{\min }} )/{\text{LAV}}_{{\max }} ] \times 100\%$$

LAEI is the relative amount of blood ejected from the LA during LV diastole relative to LA volume at start of LV systole (LAV_min_):

$${\text{LAEF = [(LAV}}_{{\max }} - {\text{LAV}}_{{\min }} )/{\text{LAV}}_{{\min }} ] \times 100\%$$

Right ventricular (RV) function was assessed by three parameters: RV two-dimensional fractional area change (RV FAC), tricuspid lateral annular systolic velocity wave velocity (RV S’) and tricuspid annular plane systolic excursion (TAPSE). RV FAC was measured by tracing the endocardial borders of the RV in a RV-focused apical four-chamber view in systole and diastole and calculated as: RV FAC (%) = 100 x (RV end-diastolic area – RV end-systolic area)/RV end–diastolic area. RV S’ was measured by tissue Doppler imaging (TDI), and TAPSE was measured by M-mode. Impaired RV function was defined as the presence of one of these three parameters; TAPSE < 17 mm, RV FAC < 35% and RV S’ <10 cm/s, as suggested by ASE guidelines [15].

We used pulsed-wave Doppler flow to measure absolute values of early diastolic flow across the mitral valve (E) and TDI of the mitral annular velocity (e’). The averaged velocity of the lateral and septal e’ was used to calculate E/e’. Pulmonary artery systolic pressure was calculated as 4 × (peak tricuspid regurgitation (TR) velocity) squared, added to the right atrial pressure estimated from the inferior vena cava diameter and collapsibility [18].

### Lung ultrasound

Eight-zone LUS examinations to assess pulmonary congestion were performed at the time of TTE, as previously described [14, 16, 19]. The total sum of B-lines in eight chest zones was used for all analyses.

### Left atrial inflow propagation velocity

**Fig. 1:**
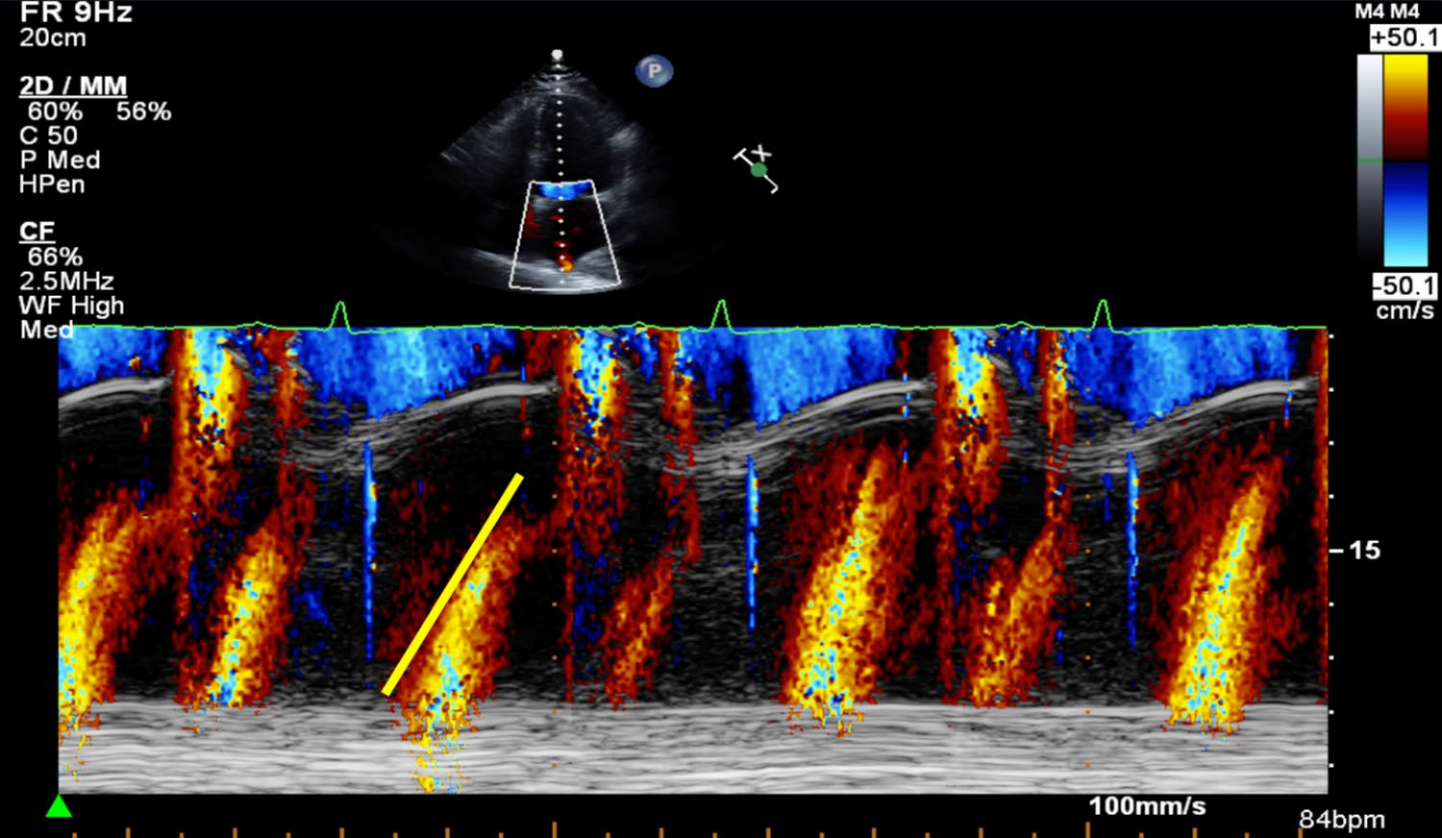
Color M-mode image demonstrating the measurement of left atrial flow propagation velocity (LAIF-PV) as the slope of the transatrial flow wave during the LA reservoir period. An illustrative example of color M-mode demonstrating the left atrial (LA) flow propagation velocity measurement. The zoom marker is placed over the LA in the apical 4-chamber view to visualize the left upper pulmonary veins outflow. After identifying the pulmonary vein jet with color Doppler, the M-mode beam is aligned to the color jet. The slope of the trans-atrial flow during the LA reservoir period (yellow line) is measured minimum 2 cm into the LA, at the color-aliasing interface or the color/no color interface of the Doppler jet

LAIF-PV was assessed from an apical four-chamber view (Fig. 1). The color Doppler sector map was adjusted to cover the entire LA, aiming to include the orifice of the right upper pulmonary vein and the mitral valve. After zooming in on the LA cavity, the M-mode cursor was placed at the center of the flow and aligned in the direction of the inflow jet. The LA inflow velocity propagation wave was measured as the linear slope of the first aliasing velocity of the flow wave of the LA from the orifice of the right upper pulmonary vein, minimum 2 cm distally into the LA cavity. The velocity scale was set to 0.5–0.7 m/s to better define the first aliasing velocity. If the slope of the transition of the first aliasing velocity could not be sufficiently defined, we measured the slope of the transition at the interface from no color to color. This method was described previously by Palecek et al., and is a modification of the methods by Brun et al. and Garcia et al. for color M-mode measurements of transmitral flow velocity [11, 12, 20]. Measurements of LAIF-PV were averaged for 1–3 consecutive cardiac cycles. Doppler velocity curves were recorded with a sweep speed set at 100 mm s^−1^ with a minimized high-pass filter. All LAIF-PV measurements were performed by a single investigator (OJ) and inter-rater agreement were assessed by a second investigator (EP) (Supplementary Material).

### Clinical outcomes

The primary clinical outcome was a composite of HF-hospitalization and all-cause mortality during 180 days after enrollment using a time-to-first event analysis [14]. Outcome data were obtained through review of the patients electronic medical records, contact with the patient’s primary care physician or cardiologist, and follow-up phone calls with the patient.

### Statistical analysis

Continuous data are expressed as mean ± standard deviation (SD) or median with interquartile range (IQR), and categorical data are expressed as counts and percentages. In the absence of established cut-off values for LAIF-PV, we divided patients into two groups based on the mean value of LAIF-PV (24.2 cm/s); a high LAIF-PV group and a low LAIF-PV group, with < 24.2 cm/s representing worse LAIF-PV. Between-group comparisons of baseline characteristics and echocardiographic data were analyzed with two sample t-test and Wilcoxon rank sum test. Categorical variables were analyzed with appropriate (Chi-square) χ^2^—tests. We used Spearman correlation and univariate linear regression to explore plausible determinants of LAIF-PV as a continuous dependent variable. We used multivariable models to adjust for potential clinical [age, sex, systolic blood pressure, heart rate, AF, body mass index (BMI), New York Heart Association (NYHA) class], and echocardiographic (LVEF, LAVi) confounders, which were selected based on prior studies of LAIF-PV [13]. Kaplan-Meier plots for the cumulative rate of HF hospitalizations or all-cause death for the high and low LAIF-PV groups were developed. We used Cox proportional hazard models to analyze the association between LAIF-PV and time to HF hospitalization or all-cause death, adjusting for clinical risk factors known to be associated with adverse outcomes in patients hospitalized for AHF; age, sex, systolic blood pressure, LVEF, LAVi and creatinine and N-terminal pro-B-type natriuretic peptide (NT-proBNP) concentrations [21]. We performed a sensitivity analysis in patients with sinus rhythm during the echocardiographic examination (excluding patients with concurrent AF; n = 7). Intra-observer and inter-observer variabilities were calculated as the absolute mean difference between the repeated measurements and 95% agreement by Bland-Altman analysis and Pearson correlation coefficient in 15 randomly selected patients with at least four weeks between the measurements. A two-sided p-value of < 0.05 was considered statistically significant for all tests. Stata SE version 14.2 (StataCorp. College Station, Texas, USA 2015) was used for all analyses.

## Results

### Study population

**Fig. 2:**
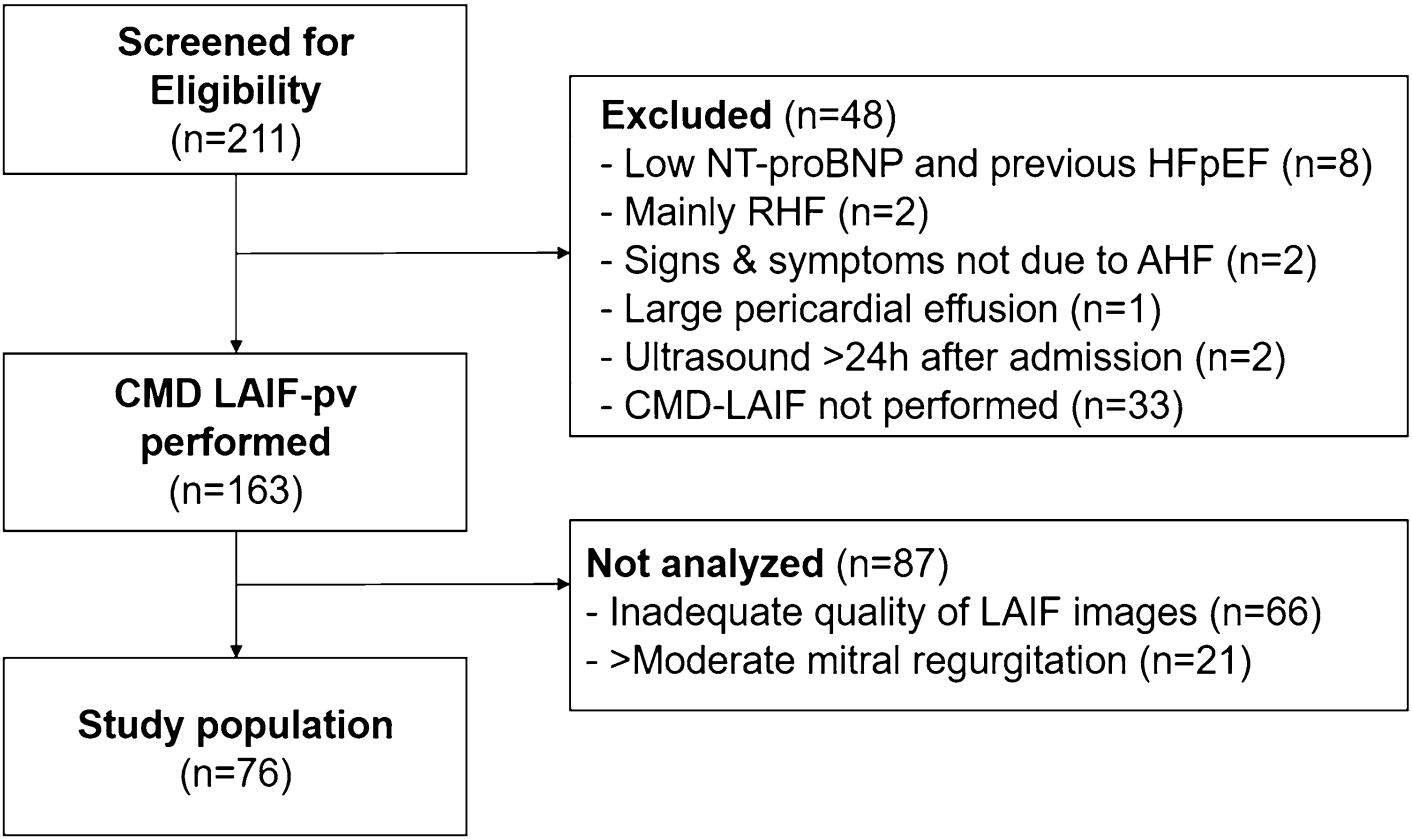
Flow chart for the study. *AHF* acute heart failure, *CMD* color M-mode Doppler, *LAIF* left atrial inflow, *MR* mitral regurgitation, *RHF* right heart failure

LAIF-PV measurements were performed in 163 (83%) of the 196 eligible patients, and 97 (60%) had adequate images for offline analysis (Fig. 2). In addition, 21 patients with moderate to severe mitral regurgitation were omitted from the analysis, yielding a study population of 76 (54%). In this study population the mean age was 71 ± 14 years, 52 (68%) were males, and 59 (78%) were white (Table 1). Patients with interpretable images for measuring LAIF-PV had a lower heart rate (75 ± 15 versus 82 ± 16 beats/min, p = 0.002) and a lower prevalence of AF (43 (39%) versus 12 (17%), p = 0.002) compared to patients who were excluded from the analysis (Suppl. Table S1**)**.

**Table 1:** Clinical characteristics of the study population (n = 76), and stratified by the mean LAIF-PV value of 24.2 cm/s

	All patients (n = 76)	LAIF-PV ≥ 24.2 cm/s (n = 35)	LAIF-PV < 24.2 cm/s (n = 41)	p-value
*Clinical characteristics*				
Age (years)	71 ± 14	71 ± 16	72 ± 13	0.70
Male sex	52 (68%)	25 (71%)	27 (66%)	0.60
White	59 (78%)	25 (71%)	34 (83%)	0.23
Body mass index (kg/m2)	27.5 (24.1–31.7)	28.6 (24.7–35.3)	26.6 (24.0–31.1)	0.19
Heart rate (bpm)	75 ± 15	75 ± 13	75 ± 16	1.00
Systolic blood pressure (mmHg)	122 ± 22	123 ± 19	122 ± 24	0.95
Diastolic blood pressure (mmHg)	67 ± 12	68 ± 14	65 ± 11	0.30
NYHA class III - IV	50 (66%)	26 (74%)	24 (59%)	0.15
*Past medical history*				
Heart failure	58 (76%)	25 (71%)	33 (80%)	0.35
Diabetes mellitus	36 (47%)	17 (49%)	19 (46%)	0.85
Hypertension	64 (84%)	29 (83%)	35 (85%)	0.76
Myocardial infarction	31 (41%)	14 (40%)	17 (41%)	0.90
PCI/CABG	35 (46%)	14 (40%)	21 (51%)	0.33
Atrial fibrillation/flutter	12 (16%)	6 (19%)	6 (16%)	0.74
Pacemaker	14 (18%)	6 (17%)	8 (20%)	0.79
CRT	6 (8%)	3 (50%)	3 (38%)	0.64
Obstructive sleep apnea	12 (16%)	3 (50%)	3 (38%)	0.64
Asthma or COPD	21 (28%)	4 (11%)	8 (20%)	0.34
*Regular medications*				
ACEi/ARB/ARNI	40 (53%)	17 (49%)	23 (56%)	0.51
Beta-blocker	62 (82%)	27 (77%)	35 (85%)	0.36
Aldosterone blocker	11 (14%)	4 (11%)	7 (17%)	0.49
Calcium channel blocker	12 (16%)	8 (23%)	4 (10%)	0.12
Insulin	19 (63%)	10 (63%)	9 (64%)	0.92
Diuretics	51 (67%)	22 (63%)	29 (71%)	0.47
Digoxin	4 (5%)	2 (6%)	2 (5%)	0.87
Warfarin/DOACs	37 (49%)	15 (43%)	22 (54%)	0.35
*Laboratory values*				
NT-proBNP (pg/ml)	5719 (3156–12,736)	4873 (2659–12,742)	7164 (3340–12,736)	0.18
Sodium (mmol/L)	137.8 ± 4.9	138.2 ± 5.1	137.5 ± 4.8	0.54
Potassium (mmol/L)	4.33 ± 0.57	4.23 ± 0.63	4.41 ± 0.50	0.17
Creatinine (mg/dl)	1.38 (1.13–2.16)	1.38 (1.07–1.78)	1.42 (1.20–2.19)	0.35
Hemoglobin (g/dl)	11.2 ± 2.0	11.0 ± 2.0	11.4 ± 2.1	0.35

*ACE* angiotensin converting enzyme, *ARB* angiotensin II receptor blocker, *ARNI* angiotensin receptor neprilysin inhibitor, *CABG* coronary artery bypass graft, *COPD* chronic obstructive pulmonary disease, *CRT* cardiac resynchronization therapy, *DOAC* Direct oral anticoagulant, *NYHA* New York heart association, *PCI* percutaneous coronary intervention, *NT-proBNP* N-terminal pro B-type natriuretic peptide

### Traditional echocardiographic and lung ultrasound measurements

Detailed results for echocardiographic measurements are presented in Table 2. The mean LVEF was 38 ± 13%, and 41 patients (54%) had heart failure with reduced ejection fraction (HFrEF) (< 40%). The mean E/e’ was 20 ± 8 and the mean E/A-ratio for patients without AF was 2.1 ± 1.1. Forty-seven (68%) had E/e’ >15, 52 (68%) had LAVi ≥ 34 ml/m^2^ and 44 (58%) had a TR velocity above 2.8 m/s. Thus, the majority (95%) had one or more echocardiographic signs of diastolic dysfunction, and the presence of diastolic dysfunction was comparable in patients with preserved and reduced LVEF.

**Table 2:** Echocardiographic measurements in the total population (n = 76), and stratified by the mean LAIF-PV value of 24.2 cm/s

	All (n = 76)	LAIF-PV ≥ 24.2 cm/s (n = 35)	LAIF-PV < 24.2 cm/s (n = 41)	P-value*
LAIF-PV (cm/s)	24.2 ± 5.9	29.2 ± 4.3	20.0 ± 3.1	–
*Lung ultrasound*				
B-lines in 8 zones (n)	10 (6–17)	12 (6–17)	10 (4–16)	0.22
*Left ventricle*				
LV systolic diameter (cm)	4.3 ± 1.0	4.4 ± 1.1	4.3 ± 1.0	0.74
LV diastolic diameter (cm)	5.3 ± 0.8	5.5 ± 0.8	5.2 ± 0.8	0.10
LV End Diastolic Volume index (ml/m2)	71 ± 23	74 ± 23	68 ± 21	0.26
LV End Systolic Volume index (ml/m2)	45 ± 21	47 ± 22	43 ± 21	0.49
LV mass index (g/m^2^)	126 ± 32	124 ± 30	128 ± 33	0.63
LV ejection fraction (%)	38 ± 13	39 ± 13	38 ± 13	0.74
E/A	2.1 ± 1.1	2.1 ± 1.0	2.1 ± 1.1	0.99
E/e’ average	20 ± 8	20 ± 9	20 ± 7	0.83
*LA size and function*				
LA volume index	37 (31–45)	39 (30–45)	37 (32–44)	0.68
LA diameter (cm)	4.6 ± 0.6	4.5 ± 0.6	4.6 ± 0.7	0.74
LA emptying fraction (%)	29 ± 15	32 ± 14	26 ± 16	0.14
LA expansion index (%)	48 ± 40	53 ± 36	45 ± 44	0.36
*Right heart*				
RV FAC (%)	24 ± 10	27 ± 10	21 ± 10	0.017
TAPSE (mm)	15.7 ± 6.3	17.3 ± 6.1	14.5 ± 6.2	0.07
RV S’ (cm/s)	10.3 ± 3.4	11.3 ± 3.7	9.4 ± 2.7	0.012
TR velocity (m/s)	3.0 ± 0.5	2.9 ± 0.5	3.0 ± 0.6	0.51
Estimated PASP (mmHg)	45 ± 15	45 ± 13	45 ± 17	0.96
IVC diameter, max (cm)	2.1 ± 0.4	2.2 ± 0.4	2.1 ± 0.4	0.20

*p-value for between high LAIF-PV group and low LAIF-PV group

Variables with missing data (patients with available measurements): E/e’ (n = 63), RV FAC% (n = 66), TAPSE (n = 70), RV S’ (n = 73), TR velocity (n = 74), estimated PASP (n = 69)

*LA* left atrium, *LAIF* left atrial inflow propagation velocity, *LV* Left ventricle, E/e’-ratio peak early mitral inflow velocity to peak septal early diastolic mitral annular velocity, *RV* right ventricle, *FAC* Fractional area change, *TAPSE* tricuspid annular plane systolic excursion, *TR* tricuspid regurgitation, *PASP* pulmonary artery systolic pressure, *IVC* inferior vena cava, *RV S´* RV systolic myocardial velocity

Measurements of the LA function were mean LAEF of 28 ± 15% and mean LAEI of 48 ± 40%. Both measures of LA function were worse in patients with reduced, compared to preserved LVEF: LAEF 25 ± 15% versus 33 ± 15%, p = 0.013 and LAEI 39 ± 35 versus 59 ± 44%, p = 0.032, respectively. RV systolic function was impaired in all 76 patients, as expressed by either one of three parameters: RV FAC, RV S’ and TAPSE. Estimated PASP was available in 69 (91%) patients and was moderately elevated (45 ± 15 mmHg). Seventy-three (96%) patients demonstrated any B-lines on LUS, with median number of B-lines 10 [IQR 6–17], range 0–34.

### Clinical and echocardiograpic predictors of LAIF-PV

Mean LAIF-PV in the total population was 24.2 ± 5.9 (range 10.6 to 41.0) cm/s. There were no statistically significant differences in age, sex, BMI, NYHA class, blood pressure, renal function or comorbidities in patients with LAIF-PV below mean (low LAIF-PV) and above mean (high LAIF-PV) (Table 1). Concurrent AF during echo was present in 7 (9%) of patients, and these patients were distributed equally in patients with high and low LAIF-PV: 3 (9%) versus 4 (10%), respectively. There was no significant difference in NT-proBNP levels between the LAIF-PV groups [median, Q1-Q3]: 7164 [3340 –2736] vs. 4873 [2659–12,742] ng/L, respectively, p = 0.18. Similar results were found when using LAIF-PV as a continuous variable (Suppl. Table S2).

**Fig. 3:**
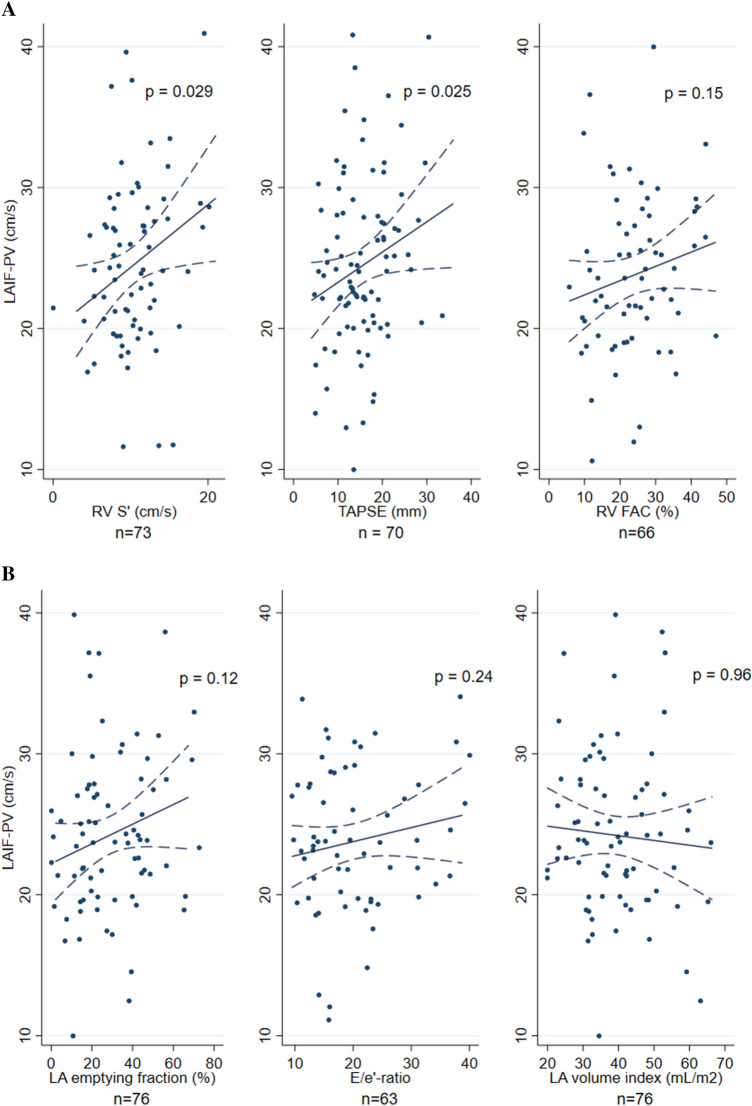
Left atrial inflow propagation velocity and measures of right ventricle (RV) systolic function. **A** RV S’ (rho = 0.29, p = 0.014), TAPSE (rho = 0.23, p = 0.05), RV FAC (rho = 0.21, p = 0.09). Scatterplots with the fitted line for the correlations between each measurement. Dotted lines indicate 95% confidence intervals. **B** Left atrial inflow propagation velocity (LAIF-PV) and measures of left atrial size and function and filling pressure. **B** LA emptying fraction (rho = 0.22, p = 0.06), E/e’ (rho = 0.07, p = 0.61), LA volume index (rho = − 0.05, p = 0.69). Scatterplots with fitted line for the correlations between each measurement. Dotted lines indicate 95% confidence intervals

Measures of RV function were worse in patients with low versus high LAIF-PV: TAPSE 14.5 vs 17.3 mm, p = 0.067, RV S’ 9.4 vs 11.3 cm/s, p = 0.012 and RV FAC 21 ± 10 vs 27 ± 10, p = 0.017 (Tables 2 and Fig. 3A). These associations were significant for TAPSE and RV S’ in regression models with LAIF-PV as a continuous variable after adjusting for age and sex (Multivariable Model 1), and additional adjustment for BMI, systolic blood pressure, heart rate, NYHA class, LVEF, (Multivariable Model 2): TAPSE β 0.28 cm/s per mm (95% CI 0.02–0.54), p = 0.039 and RV S’ β 0.46 cm/s per cm/s (95% CI 0.01–0.91), p = 0.045 but not for RV FAC (p = 0.11). (Suppl. Table S3). These associations persisted after adjusting for AF and LAVi (Multivariable Model 3). In a sensitivity analysis excluding patients with AF during echocardiography (n = 7), the associations persisted in the adjusted model for RV S’ (β 0.44, 95% CI 0.02–0.86, p = 0.039) but not for TAPSE (p = 0.18) (Suppl. Table S4). Echocardiographic measurements of LV structure and function, estimated PASP and B-lines on LUS were similar in the two LAIF-PV groups. Moreover, we found no difference in LAVi (37 vs 39 ml/m^2^, p = 0.68), E/e’ (20 vs 20, p = 0.83), or measures of LA function in patients with high versus low LAIF-PV, respectively (Table 2 and Fig. 3B). The lack of association persisted when analyzing LAIF-PV as a continuous variable in unadjusted and adjusted models (Suppl. Table S5).

### LAIF-PV and long-term outcomes

**Fig. 4:**
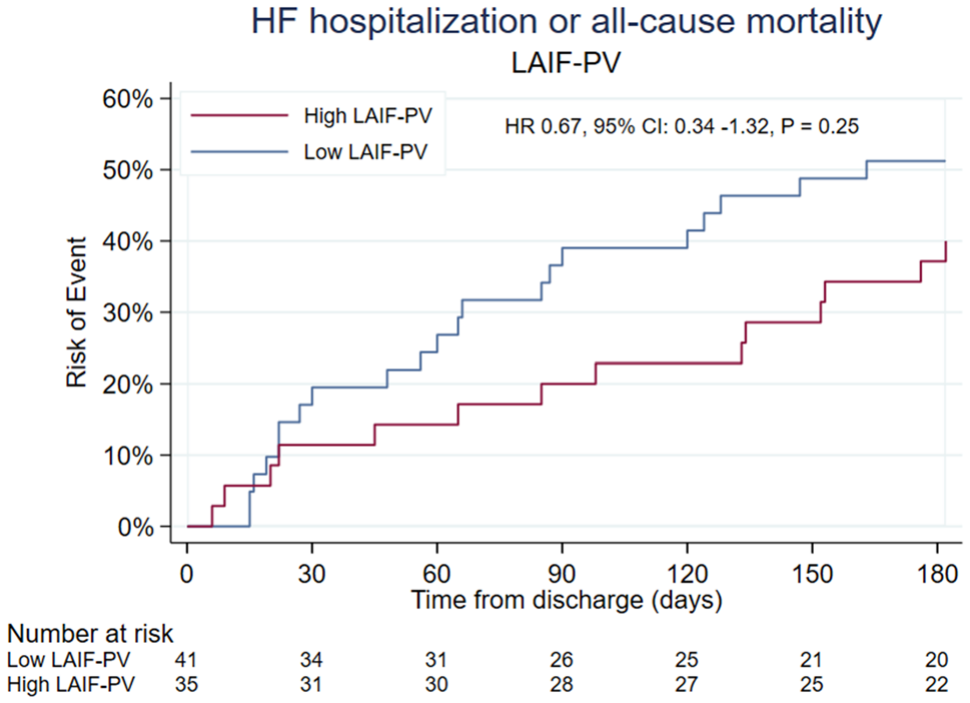
Kaplan Meyer survival curves and Cox regression analysis of high versus low left atrial inflow propagation velocity (LAIF-PV) for the primary outcome of heart failure hospitalization or all-cause mortality after enrollment

In exploratory analyses of clinical outcomes during the 180-day follow-up period after enrollment, there were 35 events, 27 hospitalizations for HF and 15 all-cause deaths **(**Table 3). Four (11%) patients in the high LAIF-PV group and 11 (27%) in the low LAIF-PV group died (p = 0.093). The number of HF hospitalizations was similar between the groups: 11 (31%) in the high LAIF-PV group vs 16 (39%) in the low LAIF-PV group (p = 0.49). Despite a numerically lower incidence of the combined primary outcome in the high LAIF group (40%) than in the low LAIF group (51%), this difference did not reach statistical significance (HR 0.67 (95% CI 0.34–1.32), p = 0.19), with consistent findings in adjusted models. LVEF, LAVi, systolic blood pressure and NT-proBNP concentrations had minimal impact on the hazard ratios and were omitted in the adjusted model to avoid overfitting. Figure 4 shows the Kaplan-Meyer curves for the primary outcome by the two LAIF-PV groups.

**Table 3:** Hospitalization for heart failure (HF) and all-cause mortality by high and low LAIF-PV groups (n = 76) during 180 day follow-up after enrollment

Primary outcome	High LAIF-PV ≥ 24.2 cm/s (n = 35)	Low LAIF- PV < 24.2 cm/s (n = 41)	p-value
Primary outcome: HF hospitalization or all-cause mortality (%)	14 (40)	21 (51)	0.33
All-cause mortality (%)	4 (11)	11 (27)	0.093
HF hospitalization (%)	11 (31)	16 (39)	0.49
Unadjusted HR for the primary composite outcome (95% CI)	Ref	0.67 (0.34–1.32)	0.25
Adjusted* HR for the primary composite outcome (95% CI)	Ref	0.69 (0.34–1.38)	0.29

*Adjusted for age, sex and creatinine

## Discussion

LAIF-PV signals were present in 83% of patients with AHF, and of these, 54% had adequate images for measuring LAIF-PV. We found no robust associations between LAIF-PV and demographics, clinical parameters, comorbidities or NT-proBNP concentrations. Lower LAIF-PV was associated with worse measures of RV systolic function. In contrast to previous studies, LAIF-PV was not associated with echocardiographic measures of LV filling pressures or LA reservoir function.

### Feasability of LAIF-PV measurements

To our knowledge, this is the first study of LAIF-PV in patients with AHF. Three prior studies evaluated the feasibility of LAIF-PV in different clinical settings, all reporting higher feasibility. Although neither of these studies are directly comparable to the present study due to differences in the patient characteristics and echocardiographic modality, they reported similar challenges in image acquisition, i.e. the inability to align the M-mode beam properly to the color jet stream or finding a distinct color M-mode slope.

Palecek et al. measured LAIF-PV with TTE under different loading conditions (leg raise, sublingual nitro) in 30 healthy adults (mean age 26 years, without known structural heart disease) [12]. They evaluated 83% of available images and found a strong correlation between LAIF-PV and LA reservoir function measures. They found LAIF-PV to be less dependent on different loading conditions than other measures of LA reservoir function (LAEI and peak systolic pulmonary venous flow velocity) and suggested that LAIF-PV is a new measure for LA reservoir function, independent of loading conditions [12]. We failed to confirm this association between LAIF-PV and markers of LA reservoir function in the present study of AHF patients.

Stoddard et al. found LAIF-PV feasible in 94% of a heterogeneous cohort of critically ill patients treated with mechanical ventilation [22]. Importantly, the LAIF-PV measurements in this study were obtained from transesophageal echocardiography (TEE). They analyzed both systolic LAIF-PV and diastolic LAIF-PV, with lower feasibility for diastolic LAIF PV (85%) [22]. The superior acoustic windows for visualizing the atria provided by TEE compared to TTE likely accounts for the higher feasibility.

Patients with interpretable images in our cohort had a significantly lower prevalence of AF and lower heart rate than non-interpretable LAIF-PV images. Prior animal and human studies have noted a diminished Doppler signal for pulmonic venous flow in the absence of effective atrial contractions, which may in part explain these differences [23]. Accordingly, measurements of LAIF-PV in AHF patients with AF seem to be particularly challenging, and thus feasibility is lower than that for patients in sinus rhythm.

### LAIF-PV measurements in AHF compared to healthy volunteers and critically ill patients

Mean LAIF-PV in our study was 24.2 cm/s, which is markedly lower than that previously reported in healthy volunteers (228 cm/s) and in critically ill patients (40 cm/s) [12, 22]. A discrepancy likely explained by different clinical settings. Mechanically ventilated patients experience elevated intrathoracic pressures from the treatment itself which likely influenced pulmonary venous flow and LAIF-PV [22]. Excluding patients with low-grade mitral regurgitation and AF may add to the heterogeneity [13]. The healthy adults from Palecek et al.’s study had a mean BMI of 22.7 kg/cm^2^, while our cohort had a higher mean BMI of 27.7 kg/cm^2^. Obesity is associated with lower feasibility for any medical imaging procedure and may have negatively impacted a highly image-quality dependent measurement such as LAIF-PV [24].

### Left atrial inflow as a measure of intracardiac pressures

Stoddard et al. found a strong correlation between LAIF-PV and invasive PCWP in critically ill patients (rho = 0.71). They also reported that LAIF-PV accurately predicts invasive PCWP within 5 mmHg, leading to great enthusiasm for this non-invasive tool for estimating LA pressures in AHF. Unfortunately, we failed to confirm these findings in patients with AHF. Although E/e’ is a suboptimal surrogate for PCWP, we expected to find a more than negligible association between the LAIF-PV and parameters for LV filling pressures [25]. The lack of association between LAIF-PV and NT-proBNP concentrations, B-lines on LUS and LA size and function strengthen our notion of a limited association between LAIF-PV and LV filling pressures in AHF. However, future studies assessing LAIF-PV in association with invasively measured PCWP in AHF are needed.

Patients hospitalized for AHF are more likely to have elevated LV filling pressures. E/e’ is commonly used as a surrogate for LV filling pressures; however, it is not without controversies. Several studies have shown the shortcomings of E/e’ in different cardiac abnormalities, and only a handful of studies have validated its feasibility and reproducibility in acute cardiac care [5]. In advanced AHF, E/e’ may not be as reliable in predicting LV filling pressures and the use of E/e’ is especially controversial in AF, with results pointing in opposite directions [26]. Most patients admitted with AHF presumably have elevated LV filling pressures as this is a key feature defining the condition. Accordingly, the lack of an association between LAIF and E/e’ may also be due to a ‘ceiling effect’, as few AHF patients actually have low or normal LV filling pressures.

Stoddard et al. studied a wider range of clinical phenotypes from septic shock to patients with prior HF. Their patients had higher mean LVEF (52%) and smaller LA diameters [22]. The clinical and cardiac structural features in patients with AHF may well affect hemodynamic parameters such as LV filling pressures and LAIF-PV, as well as the feasibility.

### Determinants of LAIF-PV in acute heart failure

The determinants of LAIF-PV are not well understood, as there are few published studies about this measure. Inflow to the LA is complex and multi-dimensional, with a flow from upper and lower pulmonary veins and backflow from mitral regurgitation during atrial contraction. It is reasonable to believe that similar forces that govern pulmonic venous flow also govern LAIF-PV. The pulsatile nature of the pulmonary venous flow and filling of the LA have been attributed to the forward transmission of RV pressure through the pulmonary vasculature tree and a suction effect, resulting from events in the LA and the LV through the cardiac cycle [23]. The latter is thought to have a higher impact on pulmonic systolic flow [23]. We found no association between demographics, vital parameters or comorbidities and LAIF-PV in the present study, in agreement with prior studies [13, 22].

Of the echocardiographic measurements, only measures of RV systolic function were associated with LAIF-PV, and persisted when adjusting for age, sex, prior HF, BMI, NYHA class, LVEF and E/e’. Palecek et al. concluded that LV and RV impact on LAIF-PV was negligible, and therefore suggested that LAIF-PV could be used as parameter of LA compliance independent of loading conditions. In contrast to healthy individuals, patients with HF have reduced LA compliance as the LA remodeling process results in larger and less compliant LA [27]. Elevated LA pressures, together with ineffective LA contractions from AF may diminish the impact of events in the LA and LV, reducing the passive “suction” effect and thus, the LAIF-PV. Furthermore, our findings may suggest that the sustained ability to propel blood through the pulmonary vascular tree by the RV may be the main driver of the LAIF-PV in AHF.

### Prognostic value of LAIF-PV in acute heart failure

In exploratory analyses of associations between LAIF-PV and outcomes, we did not find any significant difference in the combined outcome of HF hospitalization and all-cause death between patients in the high and low LAIF-PV groups. However, there was a 2-3-fold higher incidence of all-cause death in patients with lower LAIF-PV. Whether this simply reflects the well-established increased risk associated with impaired RV function, or represents LAIF-PV as an independent risk factor remains uncertain [28–30].

### Clinical application of LAIF-PV in assessing congestion

The lack of objective measures for congestion status drives a search for new tools to reliably guide intracardiac pressure evaluation. LUS has emerged as a readily available tool that is easy to learn. In this study we hypothesized that LAIF-PV would be associated with established markers of congestion. However, we found no associations between LAIF-PV and E/e’, LA size and function, LUS measures of pulmonary congestion, NYHA class, or NT-proBNP concentrations, which suggests that LAIF-PV is a poor surrogate for congestion in AHF. Nevertheless, LAIF-PV may provide insights into LA flow dynamics and RV performance in select patients with advanced HF.

### Study limitations

This was a single-center study of heterogeneous patients hospitalized with AHF, with a modest sample size. A larger sample size would allow for analysis of LAIF-PV in subgroups categorized by LVEF ≤ 40%, 41–49%, and ≥ 50%. The study lacks invasive hemodynamic measurements of LA and LV filling pressures. Due to the low event rate, we likely had insufficient power to detect a difference in outcomes between the two groups. Future investigations should validate LAIF-PV against invasive- or non-invasive LA function measurements, such as cardiac magnetic resonance imaging or echocardiography with deformation analysis. We did not evaluate pulmonary venous flow by Doppler. Although Color-M-mode provides excellent time and spatial relationships to map the velocity change of blood flow, any two-dimensional method is unlikely to explain the complex multi-dimensional flow properties of the LA. The inter-observer variability of the LAIF-PV measurements was 0.70, and automated tracing by dedicated software and standardization of the measurements may improve the reliability of LAIF-PV measurements in future studies [31, 32].

## Conclusions

Color M-mode derived LAIF-PV is a technically challenging measurement. In this cohort of AHF patients, lower LAIF-PV was associated with echocardiographic signs of RV systolic dysfunction. There were no associations between LAIF-PV and established clinical, biomarker, echocardiographic and LUS markers of congestion. Future studies should examine the association between LAIF-PV and invasive hemodynamics in patients with and without HF.

### Supplementary Information

Below is the link to the electronic supplementary material.

Supplementary material 1 (DOCX 31.4 kb)

## Data Availability

Due to the nature of this research, participants of this study did not agree for their data to be shared publicly.
