# Supplementary material for: Left atrial inflow propagation velocity derived by color M-mode Doppler in acute heart failure
Source: Int J Cardiovasc Imaging. 2022 Apr 23;38(10):2155–65. doi: 10.1007/s10554-022-02614-y (PMC10247843; doi:10.1007/s10554-022-02614-y)
Supplement: Supplementary file 1 — Supplementary material 1 (DOCX 31.4 kb) [file 10554_2022_2614_MOESM1_ESM.docx]

# Supplementary Material

to

# Left Atrial Inflow Propagation Velocity derived by Color M-Mode Doppler in Acute Heart Failure

## Intra- and inter-observer agreement

The mean difference in intra-observer agreement for LAIF-PV measurements was -0.66 cm/s (95% limits of agreement -8.9 to 7.7) with averages between 19.7 cm/s and 38.7 cm/s and the correlation coefficient was 0.74 and (p = 0.002). The mean difference in inter-observer agreement in LAIF-PV measurements was -5.8 cm/s (95% limits of agreement, -21.2 to 9.6) and the correlation coefficient was 0.70 (p = 0.004).

## Table S1. Differences in selected baseline and echocardiographic characteristics for patients with measurable LAIF-PV (n = 76) and patients who were excluded or had unmeasurable LAIF-PV (n = 120)

|  | **Included**  **(n = 76)** | **Excluded**  **(n = 120)** | **P-value** |
| --- | --- | --- | --- |
| Age (years) | 71 ± 15 | 70 ± 15 | 0.66 |
| Male | 52 (68%) | 68 (57%) | 0.10 |
| Body mass index [kg/m2] | 27.5 [24.1-31.7] | 27.7 [23.7-34.1] | 0.68 |
| Heart Rate (bpm) | 75 ± 15 | 82 ± 16 | 0.002 |
| Chronic Heart Failure | 58 (76%) | 91 (76%) | 0.94 |
| Myocardial Infarction | 31 (41%) | 34 (28%) | 0.071 |
| Mitral Regurgitation | 13 (17%) | 36 (30%) | 0.042 |
| Prior Atrial fibrillation/flutter | 12 (17%) | 43 (39%) | 0.002 |
| Atrial fibrillation/flutter during echocardiogram | 7 (9%) | 27 (23%) | 0.017 |

## Table S2. Association between LAIF-PV as a continuous dependent variable with clinical, laboratory and hemodynamic values

|  | **ß** | **(95%CI)** | **P-value** |
| --- | --- | --- | --- |
| Age (per 5 years) | -0.25 | (-0.72 to 0.22) | 0.29 |
| Male Sex | -0.91 | (-3.8 to 2.0) | 0.54 |
| Body mass index (per 5 kg/m^2^) | 0.47 | (-0.54 to 1.48) | 0.36 |
| New York Heart Association class III-IV | 1.21 | (-1.6 to 4.1) | 0.40 |
| Obstructive pulmonary disease | 2.82 | (-0.1 to 5.8) | 0.06 |
| Atrial fibrillation/flutter | 1.41 | (-2.4 to 5.3) | 0.47 |
| Heart Rate (per 10 bpm) | 0.24 | (-0.69 to 1.18) | 0.60 |
| Systolic blood pressure (per 10 mm hg) | 0.14 | (-0.48 to 0.77) | 0.65 |
| Creatinine | 0.58 | (-2.0 to 0.8) | 0.40 |
| Log N-terminal pro-B-type natriuretic peptide (pg/mL) | 0.94 | (-2.3 to 0.4) | 0.17 |

## Table S3. Univariate and multivariable linear regression analysis of LAIF-PV in association to measures of right ventricular function

|  | **Univariable** | **Multivariable Model 1**  (age, sex) | **Multivariable Model 2**  (Model 1 + BMI, systolic BP, heart rate, NYHA-class and LVEF) | **Multivariable Model 3**  (Model 2+ afib_during echo and LAVi) |
| --- | --- | --- | --- | --- |
| **RV measures** |  |  |  |  |
| TAPSE (mm) (n = 70) | 0.26  (0.03 to 0.48)  P = 0.025 | 0.26  (0.03 to 0.49)  P = 0.026 | 0.28  (0.02 to 0.54)  P = 0.039 | 0.27  (0.01 to 0.53)  P = 0.041 |
| RV S’(cm/s) (n = 73) | 0.45  (0.05 to 0.85)  P = 0.029 | 0.44  (0.04 to 0.85)  P = 0.031 | 0.46  (0.01 to 0.91)  P = 0.045 | 0.47  0.03 to 0.92  P = 0.035 |
| RV FAC (per 5% increase) (n=66) | 0.51  (-0.19 to 1.20)  P = 0.15 | 0.54  (-0.17 to 1.25)  P = 0.13 | 0.64  (-0.15 to 1.43)  P = 0.11 | 0.71  -0.1 to 1.51  P = 0.084 |

*Presented as ß and 95% CI*

**Abbreviations:**TAPSE = tricuspid annular plane systolic excursion, RV S´= RV systolic myocardial velocity, FAC = Fractional Area Change

## Table S4. Univariate and multivariable regression analysis of LAIF-PV in association to measures of right ventricular function, excluding patients with atrial fibrillation during echocardiography

|  | **Univariable** | **Multivariable Model 1**  (age, sex) | **Multivariable Model 2**  (Model 1 + BMI, systolic BP, heart rate, NYHA-class and LVEF) | **Multivariable Model 3**  (Model 2+ LAVi) |
| --- | --- | --- | --- | --- |
| TAPSE (mm) (n = 63) | 0.15  (-0.07 to 0.37)  P = 0.187 | 0.17  (-0.05 to 0.4)  P = 0.13 | 0.18  (-0.09 to 0.45)  P = 0.18 | 0.18  (-0.08 to 0.45)  P = 0.18 |
| RV S’(cm/s) (n = 66) | 0.40  (0.03 to 0.76)  P = 0.033 | 0.42  (0.05 to 0.79)  P = 0.026 | 0.44  (0.02 to 0.86)  P = 0.039 | 0.43  (0.01 to 0.85  P = 0.043 |
| RV FAC (per 5%) (n=60) | 0.63  (-0.03 to 1.29)  P = 0.062 | 0.71  (0.04 to 1.39)  P = 0.037 | 0.75  (-0.01 to 1.51)  P = 0.054 | 0.75  (-0.01 to 1.52)  P = 0.053 |

*Presented as ß and 95% CI*

**Abbreviations:**TAPSE = tricuspid annular plane systolic excursion, RV S´= RV systolic myocardial velocity, FAC = Fractional Area Change

**Table S5. Univariate and multivariable regression analysis of the association between LAIF-PV and measures of left atrial structure and function and number of B-lines on lung ultrasound**

|  | **Univariable** | **Multivariable Model 1**  (age, sex) | **Multivariable Model 2**  (Model 1 + BMI, systolic BP, heart rate, NYHA-class and LVEF) | **Multivariable Model 3**  (Model 2+ afib during echo LAVi) |
| --- | --- | --- | --- | --- |
| LAVi (mL/m2) | -0.03  (-0.16 to 0.09)  P = 0.59 | -0.008  (-0.14 to 0.13)  P = 0.90 | -0.004  (-0.15 to 0.14)  P = 0.95 | -0.03  (-0.18 to 0,11)  P = 0.63 |
| LAEF (per 5 %) | 0.70  (-0.19 to 0.16)  P = 0.12 | 0.06  (-0.03 to 0.16)  P = 0.21 | 0.06  (-0.05 to 0.17)  P = 0.26 | 0.09  (-0.02 to 0.22)  P=0.12 |
| LAEI (%) | 0.02  (-0.01 to 0.05)  P = 0.23 | 0.02  (-0.02 to 0.05)  P = 0.37 | 0.02  (-0.02 to 0.06)  P = 0.45 | 0.02  (-0.02 to 0.07)  P = 0.29 |
| Sum of B-lines across 8 zones (per 3 lines) | -0.1  (-0.66 to 0.46)  P = 0.73 | -0.05  (-0.62 to 0.51)  P = 0.85 | -0.04  (-0.66 to 0.58)  P = 0.91 | -0.04  (-0.66 to 0.57)  P = 0.88 |

*Presented as ß and 95% CI*

**Abbreviations:**LAVI = left atrial volume index; LAEF = left atrial emptying fraction; LAEI = left atrial expansion index
